# Development of viral infectious clones and their applications based on yeast and bacterial artificial chromosome platforms

**DOI:** 10.1186/s43556-025-00266-7

**Published:** 2025-04-29

**Authors:** Yiyi Wu, Shangqing Gao, Guanya Liu, Mengwei Wang, Ruixiao Tan, Baoying Huang, Wenjie Tan

**Affiliations:** 1 National Key Laboratory of Intelligent Tracking and Forecasting for Infectious Diseases, NHC Key Laboratory of Biosafety, National Institute for Viral Disease Control and Prevention, China CDC, 155 Changbai Road, Beijing, 102206 China; 2 School of Public Health, Baotou Medical College, Baotou City, Inner Mongolia Autonomous Region 014040 China; 3 College of Life Sciences, Beijing Normal University, 19 Xinjiekouwai Avenue, Beijing, 100875 China

**Keywords:** Virus Infectious Clones, Reverse genetics, Yeast artificial chromosome (YAC), Bacterial artificial chromosome (BAC), Application

## Abstract

Infectious Clones represent a foundational technique in the field of reverse genetics, allowing for the construction and manipulation of full-length viral genomes. The main methods currently used for constructing viral infectious clones include Transformation-associated recombination (TAR), which is based on Yeast Artificial Chromosome (YAC) and Bacterial Artificial Chromosome (BAC). The YAC and BAC systems are powerful tools that enable the clones and manipulation of large DNA fragments, making them well-suited for the construction of full-length viral genomes. These methods have been successfully applied to construct infectious clones for a wide range of viruses, including coronaviruses, herpesviruses, flaviviruses and baculoviruses. The rescued recombinant viruses from these infectious clones have been widely used in various research areas, such as vaccine development, antiviral drug screening, pathogenesis and virulence studies, gene therapy and vector design. However, as different viruses possess unique biological characteristics, the challenge remains in how to rapidly obtain infectious clones for future research. In summary, this review introduced the development and applications of infectious clones, with a focus on the YAC, BAC and combined YAC-BAC technologies. We emphasize the importance of these platforms in various research areas and aim to provide deeper insights that can advance the platform and broaden its application horizons.

## Introduction

Recent breakthroughs in DNA synthesis have unlocked unprecedented opportunities in various fields, including medicine, industry, agriculture, and research. Synthetic genomics involves the engineering of viruses, bacteria, and eukaryotic cells from artificial genomes, with its primary scope concentrating on the synthesis of entire genomes or chromosomes and the later animation of these synthetic nucleic acids to yield viruses or living cells. This field has notably influenced reverse genetics of viruses and propelled vaccine design and production [1, 2]. Reverse genetics is a technique that allows live viruses to be rescued through cDNA cloning of the virus. This innovative technique enables scientists to systematically modify the viral genome sequence under laboratory conditions, allowing them to study the viral gene structure, function, and the interaction between the virus and the host [3–5]. As the core of reverse genetics, infectious clone is a laboratory-constructed DNA copy of a virus that can be used to produce live, infectious virus particles when introduced into host cells [6]. Beginning with the development of the first infectious clone for poliovirus [7], a wide range of virus infectious clones have now been rescued, including coronaviruses [1, 8, 9], herpesviruses [10, 11], flaviviruses [12–14], baculovirus [15], rhabdoviruses [16] and Lassa virus [17].

**Fig. 1:**
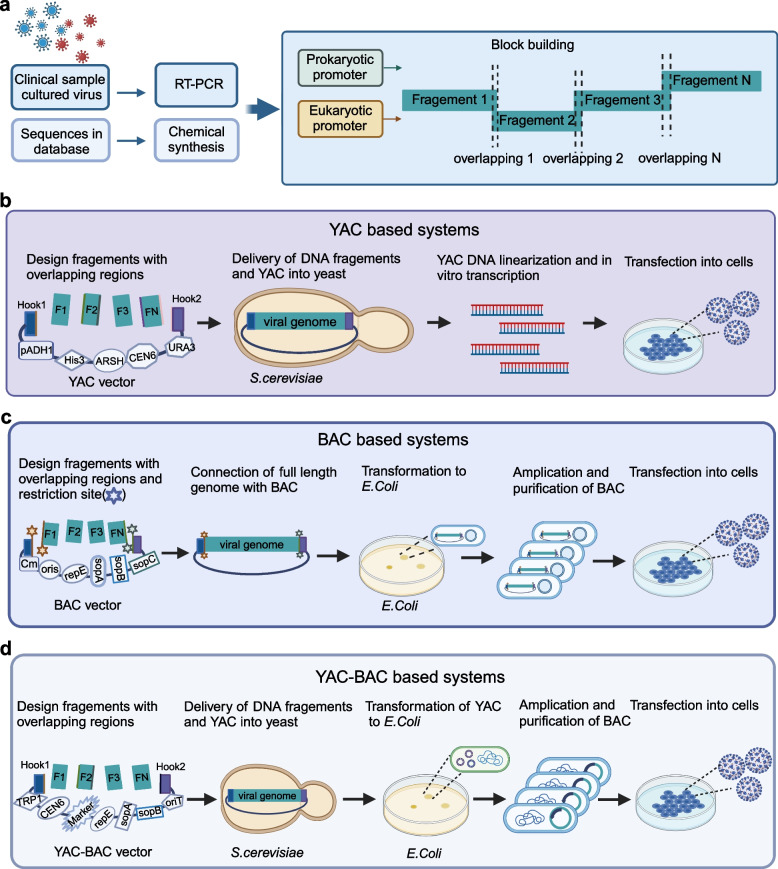
Overview of the strategies and processes involved in constructing viral infectious clones using Yeast Artificial Chromosome (YAC), Bacterial Artificial Chromosome (BAC), and YAC-BAC combined systems. **a** The full-length viral genome is amplified from clinical isolated viruses or sequence databases by PCR or chemical synthesis. Then the genome is divided into overlapping fragments and ligated with a promoter for later experiments. **b** YAC based systems. Overlapping DNA fragments were delivered into *S.cerevisiae* yeast along with a linearized YAC/TAR vector. Homologous recombination in the yeast cells assembles the DNA fragments to generate the YAC vector containing the viral full-length cDNA. The infectious clones are then produced in vitro by plasmid linearization and run-off T7 RNA polymerase-based transcription. Virus rescue is initiated by transfection of RNA into susceptible cells, followed by virus production and amplification. **c** BAC based systems. Restriction endonucleases were used to insert the DNA fragments into a BAC vector, and ligation to generate the viral full-length cDNA. The recombinant constructs are transformed into *E. Coli* for selective cultivation, and positive transformants are amplified and transfected into host cells for virus rescue. **d** YAC-BAC systems. Overlapping DNA fragments are delivered into yeast cells along with a linearized YAC vector, and all DNA fragments are assembled by homologous recombination to generate the YAC plasmid containing the viral full-length cDNA. The YAC plasmid is then transformed into *E. Coli* to produce a BAC plasmid, which is subsequently used to rescue recombinant viruses by transfecting it into susceptible cells

Various methodologies have used for infectious clone construction, such as in vitro ligation [13, 18, 19], transformation-associated recombination (TAR) with Yeast Artificial Chromosome (YAC) [20], Bacterial Artificial Chromosome (BAC) technologies [3, 21, 22], the integration of YAC-BAC approach [12, 23, 24], Circular Polymerase Extension Reaction (CPER) [25–27] and Infectious Subgenomic Amplicons (ISA) [14, 28]. YAC is an engineered vector system designed to replicate like a chromosome and stably maintain large DNA fragments within yeast cells. BAC is DNA construct used to clone large DNA sequences in bacteria, most commonly *Escherichia coli* (*E. coli*), BACs are based on naturally occurring plasmids, such as the F-plasmid, which can stably maintain large DNA inserts. The integration of methodologies, particularly YAC and BAC systems, has greatly enhanced the ability to assemble and study complex viral genomes. The emergency of Severe Acute Respiratory Syndrome Coronavirus 2 (SARS-CoV-2) has significantly accelerated the application of viral cloning technologies. The infectious clones’ templates can be derived from clinical isolation samples or viral genome sequences in databases, breaking through the limitation of relying on live viruses [20, 29]. In practice, the viral genome sequence can be divided into several fragments for later homologous recombination. For the YAC based strategy, whole genome fragments are ligated to a linearized YAC vector, and YAC plasmid containing the viral full-length cDNA is obtained with the efficient homologous recombination mechanism of yeast, the YAC plasmid is ultimately transfected into sensitive cells for virus rescue. For the BAC strategy, restriction enzyme sites and other methods are used to connect the full-length viral genome with the BAC vector, then is transformed into *E. coli* for amplification, the purified BAC plasmid is further transfected into sensitive cells. For the YAC-BAC combined technology, viral genome fragments are delivered into yeast cells along with a linearized YAC-BAC shuttle vector. The plasmid is electroporated into *E.coli* for amplification, and then transfected into sensitive cells to rescue the virus (Fig. 1).

Infectious clone technology has played an important role in biomedicine. Shortly after the emergence of COVID-19, researchers from the University of Bern in Switzerland obtained recombinant SARS-CoV-2 virus using infectious clones technology within one week, providing tools for rapid virus detection and vaccine development [1]. Researchers from the University of Texas Medical Branch used synthetic biology and BAC to construct infectious cDNA of the novel coronavirus, and successfully generated SARS-CoV-2 virus using a trans-complementing cell line, enabling biosafety laboratory level 2 (BSL-2) experimental operations [30]. Additionally, a research team from the University of North Carolina revealed the infection gradient and pathogenic mechanisms of SARS-CoV-2 in the respiratory tract using a reverse genetics system [31]. In clinical medicine, infectious clones technology has also played a significant role. Kanai and colleagues used the VP4 and VP7 genes from human rotavirus clinical samples as scaffolds, inserting them into the rhesus monkey rotavirus SA11 strain to rapidly construct a candidate vaccine matching the current circulating viral genotype, supporting the production of viral antigenic tests and diagnostic tools [32]. By constructing defective viral particles, researchers were able to simulate the complete viral lifecycle under biosafety conditions, thereby screening and testing antiviral drugs [17]. Oncolytic viruses is engineered or naturally occurring viruses that selectively infect and lyse cancer cells while sparing normal tissues [33–35], by inserting immune-modulatory genes, they can reshape the tumor microenvironment to enhance anti-tumor immune responses and improve treatment outcomes [36].

The aim of this review is to describe the mechanisms underlying the construction of infectious clones using YAC, BAC, and YAC-BAC technologies, along with construction strategies for various viruses. We also summarize the applications of infectious clone platforms in vaccine development, antiviral drug screening, pathogenetic research, gene therapy, and vector construction. The objective is to offer deeper insights into infectious clone technology and broaden its scope of applications.

## Yeast artificial chromosome recombination technology

Yeast Artificial Chromosome (YAC) technology is a powerful tool for cloning and manipulating large DNA fragments. With the efficient homologous recombination system, viral genomes could be precisely assembled or modified, making the YACs a robust platform for virus infectious clones’ construction. At present, YAC technology has been widely applied to constructing infectious clones for various viruses with reporter genes or attenuate characteristics, improving our understanding of viral pathogenesis and accelerating biomedicine research.

### Mechanism and Development of YAC system

In 1983, Murray and Szostak initiated the Yeast Artificial Chromosomes (YACs) technology by integrating key yeast elements—centromere (*CEN4*), Autonomous Replication Sequence 1 (ARS1), and Tetrahymena ribosomal RNA termini (Tr)—into the pBR322 plasmid of *E. coli*. The unique yeast centromere, *CEN4*, advocated for normative cell division, while the *ARS1* sequence provided a replication origin. Additionally, the Tetrahymena ribosomal RNA termini sequence, following *BamH*I digestion, formed terminal structures resembling telomeres, which help prevent chromosomal attrition and unwanted recombination [37].

The mechanism of YAC primarily based on the homologous recombination system in yeast, especially for fragment repair and ligation [38]. This process including the DNA double-strand break (DSB) formation, end resection, homologous sequence pairing, strand invasion, DNA synthesis and extension, the formation and resolution of Holliday junction, as well as repair synthesis and ligation [39, 40]. The accuracy and efficiency of recombination are mainly mediated by serial Rad proteins such as *Rad51*, *Rad52*, *Rad54*, and *Mus81-Mms4* [41].

Yeast’s efficient recombination repair machinery facilitated the development of transformation-associated recombination (TAR). The efficacy of TAR cloning depends on several factors, including the length and specificity of the overlapping sequence, as well as the presence of other unique sequences [42–44]. TAR vectors typically incorporate a YAC cassette to manage gene expression in yeast, a BAC cassette to ensure replication stability in bacterial cells, flanked by specialized sequences at both ends of the target fragment. The YAC cassette regulates the expression, isolation, and selection of the target gene in yeast. The BAC cassette contains a bacterial replication origin and selection markers, enabling TAR products to replicate and remain stable in bacterial cells, thus supporting large-scale DNA fragment amplification in bacteria. TAR vectors use an Autonomous Replication Sequence (*ARS*) as the replication origin in yeast. The specialized 'hooks' at the termini of the TAR vector represent overlapping sequences that guide the precise insertion of the target fragment. Research indicates that an overlap as minimal as 15 bp is efficient for facilitating homologous recombination [45], while a 30 bp overlap can ensure an 80% success rate [46, 47]. The co-transformation of the vector and target fragment in yeast triggers homologous recombination, driven by the yeast’s innate repair capabilities, culminating in the seamless integration of the fragments [48, 49].

### Advantages of YAC technology in viral genomics

YACs can harbor large DNA fragments, typically spanning 200 to 500 kb, and in some cases up to 1 Mb in size [50]. These vectors are particularly suitable for constructing infectious virus clones because of their lack of viral DNA restriction sites, thus allowing for full genomic encapsulation. Additionally, since the creation of infectious clones occurs in yeast, the time-consuming plaque purification step of recombinant virus is eliminated [51]. However, challenges such as chimerism, potential clone instability with passage, and occasional deletions and rearrangements must be considered [52, 53].

TAR leverages homologous recombination enabling precise gene isolation and recombination in *Saccharomyces cerevisiae* (*S. cerevisiae*), thereby eliminating the need for labor-intensive random genomic YAC and BAC library construction and screening. By designing homologous overlapping sequences across multiple fragments, TAR facilitates one-step genome assembly [54, 55]. This method was exemplified by the work of Thao et al., who rescued SARS-CoV-2 by incorporating a reporter gene and Mouse Hepatitis Virus (MHV A59) using an in vitro transcription system with the T7 promoter [1]. Furthermore, this approach enabled the precise cloning of Human coronavirus HKU1 (HCoV-HKU1) [56] and Zika virus (ZIKV) [57]. Crucially, TAR cloning reduced the reliance on restriction enzyme sites, enhancing precision without incorporating extraneous sequences and streamlining the concurrent construction and transformation of plasmids.

### Challenges with YAC technology

The limitations of using TAR in yeast for constructing infectious virus clones include issues with DNA repair mechanisms, recombination efficiency, YAC extraction and purification, and the error-prone in vitro transcription step, as well as the lower transfection efficiency of RNA.

DNA repair mechanisms such as non-homologous end joining (NHEJ) and microhomology-mediated end joining (MMEJ) can significantly impair the efficacy of TAR [58]. Factors such as high GC content [59] and UV treatment [60] may also reduce the efficiency of yeast recombination processes.

The process of extracting yeast plasmids is complex and time-consuming. In theory, pulsed-field gel electrophoresis can be used to separate circular YACs from linearized yeast chromosomes, but this method is time-consuming, yields low quantities, and low throughput. Additionally, the concentration of yeast obtained is insufficient to meet the requirements for transfection [61]. Devenish et al. developed a method for purifying ~ 200 kb circular plasmids from yeast chromosomes III via alkali extraction, but the reproducibility of this method is poor, and the quality of the DNA is low [62]. Noskov et al. improved this approach, enabling the isolation of high-quality microgram-level YAC DNA. However, this method requires at least three days to obtain the plasmid, and careful handling is needed at each step to achieve high-purity DNA [63].

YAC technology requires generating viral RNA genomes from YAC DNA plasmids through in vitro transcription, followed by transfecting the RNA into susceptible cells [64]. However, in vitro transcription with T7 and SP6 polymerases have high error rates, with approximately 2 errors per 10 kb for the T7 promoter [65] and 1 error per 10 kb to 30 kb for the SP6 promoter [66]. The additional in vitro transcription step, along with the relatively low electroporation efficiency of RNA compared to DNA transfection, indicates that optimizing this system is necessary [9].

### Examples of viral infectious clones developed using YAC technology

Compared to bacteria, yeast displays reduced sensitivity to viral toxic sequences and demonstrates superior capacity for maintaining large DNA fragments [51]. This characteristic has contributed to its growing popularity in the fields of synthetic genomics and reverse genetics. The development of virus infectious clones utilizing YAC dates back to 1994, when researchers at Johns Hopkins University successfully constructed an infectious clone containing the complete genome of human adenovirus type 2 (HAdv 2) by employing homologous recombination between adenoviral DNA and the YAC vector pRML, which included viral end sequences [51]. This approach was subsequently refined and optimized, leading to the establishment of TAR [67]. TAR was originally developed for the isolation of eukaryotic DNA fragments from yeast. It was later adapted for constructing large double-stranded DNA viruses [20, 24] and for assembling entire bacterial genomes, such as those of mycoplasmas (~ 1 Mb) [68, 69]. With the advancement of synthetic biology, TAR technology has been successfully employed for the rapid reconstruction of various RNA viruses, including coronaviruses [1, 8] and flaviviruses [70, 71], as well as DNA viruses from families such as herpesviruses [72], poxvirus [73], asfarvirus [74] and alphavirus [75].

Thao et al. used TAR to reconstruct the SARS-CoV-2 genome within one week. The process involved amplifying the viral genome cDNA via reverse transcription polymerase chain reaction (RT-PCR) and chemical synthesis, followed by segmentation into 12 overlapping fragments. To facilitate transcription by T7 polymerase in vitro, a T7 promoter was added to the 5'end, and *Eag*I restriction sites were incorporated at the 3'polyadenylated (polyA) tail. The cDNA fragments, along with the linearized TAR vector pCC1BAC-His3, were co-transformed into *S.cerevisiae*, enabling the assembly of the full-length viral cDNA through the yeast's efficient homologous recombination and repair mechanisms. Following purification and digestion of positive clones, linearized YAC plasmids were obtained and used for in vitro synthesis of capped viral genomic RNA with T7 RNA polymerase. The synthesized RNA was then co-electroporated with mRNA encoding the SARS-CoV-2 nucleocapsid protein into BHK-21 cells. Following electroporation into Vero E6 cells, SARS-CoV-2 was successfully rescued. A GFP reporter gene was inserted into the ORF7a frame, resulting in recombinant viruses that carried the reporter gene. Comparative analysis of viral replication in Vero E6 cells showed that both the parental SARS-CoV-2 isolate, and the recombinant had similar replication profiles. However, the recombinant virus carrying the GFP reporter gene demonstrated reduced replication relative to the wild-type recombinant virus [1]. TAR is noted for its rapidity and versatility, rendering it an appealing approach for swiftly addressing new viral threats. While this technique streamlines the targeted assembly process, it is imperative to perform sequencing to ensure that no unintended mutations have been introduced into the yeast plasmids during the homologous recombination and propagation phases in yeast [69, 76, 77].

## Bacterial artificial chromosome recombination technology

Although the YAC base technology is suitable for cloning large DNA fragments, the technical complexity such as multiple-steps and low plasmid yield, has limited its application in virology. In contrast, Bacterial Artificial Chromosome (BAC), with easy manipulation and high stability, has emerged as an important tool for constructing infectious clones.

### Mechanism and development of BAC system

The initial prototype BAC vector, pBAC108L, was engineered by integrating a minimized F factor from *E. coli*, which encompassed the replication and partitioning genes *oriS* and Replication protein E(repE), along with Partitioning protein A (*parA*) and *parB* for stringent copy number control, and a chloramphenicol resistance (CMR) gene for selection purposes [78]. To address the absence of recombinant selection markers in the original constructs, researchers added the *lacZ* gene fragment, thus generating the enhanced vector, pBeloBAC11 [79]. The second-generation BAC supports the incorporation of large DNA fragments and demonstrates higher propagation efficiency in recombinant-deficient *E. coli* strains, significantly surpassing performance metrics of yeast-based systems [80]. pBeloBAC11 provides substantial stability for exogenous viral sequences, facilitating both plasmid amplification and the reduction of viral sequence toxicity [81]. This platform has been effectively used in research on coronaviruses and herpesviruses [3, 18, 82, 83]. Despite its advantages, limitations such as low copy number and constrained DNA yield remain barriers to broader application. Recent innovations have spurred the development of new BAC series vectors derived from the second-generation models [84–86], significantly advancing synthetic genomics efforts. Frijter et al. has enhanced the selection markers in BAC vectors by introducing a spectinomycin resistance marker [87]. Baker et al. crafted BAC vectors with a reporter gene by cloning a cytomegalovirus (CMV) promoter-driven fluorescent reporter gene into these vectors [88]. Additionally, the high-copy replication BAC vector pCC1BAC [89], and the pSMART BAC [90–92], have been employed effectively in constructing viral infectivity clones.

### Advantages of BAC technology in virology

Bacterial Artificial Chromosomes (BACs), initially conceived by Shizuya et al. in 1992, represent complex vector systems derived from the F plasmid of *E.coli*, effectively simulating natural chromosomal mechanisms BACs are instrumental due to their capacity to maintain low per-cell copy numbers stably and can contain DNA fragments that range from 150 to 350 kilobases in length [78]. Characterized by a reduced propensity for chimeric anomalies and structural rearrangements, BACs function as self-replicating units capable of housing complete viral cDNAs [54]. Additionally, the incorporation of the CMV immediate-early promoter facilitated direct initiation of viral RNA synthesis within target cells, obviating the need for RNA synthesis in vitro [93].

### Challenges with BAC technology

There are several challenges for BAC-based reverse genetics technologies. Firstly, virus rescue in cell cultures, which can induce adaptive mutations in viral genomes, compromising their genetic integrity [94]. Additionally, the traditional screening methods for identifying and recovering recombinants may inadvertently select for viral strains adapted to cell culture conditions rather than those present in clinical samples, introducing a selection bias that limits the technology's application in directly cloning viral genomes from such samples [20, 95].

Moreover, the presence of viral repetitive sequences can result in phenotypic discrepancies when amplifying BAC clones in *E. coli* [96]. The complete sequencing of the viral genome is necessary to locate suitable restriction sites for cloning, a step complicated by the absence of unique restriction sites in many viruses. Assembled genomes using BAC vectors are prone to instability [97, 98], and sequences susceptible to deletion during replication in *E. coli* due to their repetitive nature [94, 99].

The homologous recombination used to integrate the viral genome with the BAC vector can expose the genomic sequence to recombinases, risking the introduction of mutations [97, 100]. Furthermore, each modification of the genome necessitates sequential execution, significantly extending the timeline of the experiment [24]. In the case of RNA viruses, such as coronaviruses, the viral cDNA may have toxicity to *E. coli* [101], requiring the adoption of strategies like synonymous mutation, insertion of intron sequences, or in vitro ligation to reduce toxicity to *E. coli* [8]. Rigorous verification of the viral sequence within the plasmid is imperative at each stage to prevent unintended mutations during bacterial propagation [55].

### Examples of viral infectious clones developed using BAC technology

Bacterial Artificial Chromosomes (BACs) are highly favored in the field of virology due to their large cloning capacity and replication fidelity, making them particularly useful for constructing infectious clones of viruses such as herpesviruses and coronaviruses. In 1997, Messerle et al. first use of BAC technology for constructing a first full-length infectious clone of mouse cytomegalovirus (MCMV), thereby setting the first viral BAC clone [102]. In this system, researchers used homologous recombination to integrate the pKSO vector and the gpt selection marker into a non-essential region of the virus’s episomal replication cycle, followed by cloning and screening of the complete MCMV DNA in *E. coli* CBTS. The plasmid containing the virus was then transfected into eukaryotic MEF cells for the rescue of recombinant viruses. This method facilitates the introduction of multiple mutations through successive experimental iterations without necessitating the reconstruction of infectious virus intermediates. The approach of conducting additional rounds of mutagenesis in *E. coli* simplifies the construction of infectious clones. Given the relatively slow replication rate of BACs and the ease and precision of generating mutants, BAC technology has been effectively used in herpesvirus research. Furthermore, Guo et al. used CRISPR/Cas9 technology to optimize the construction of bacterial artificial chromosome (BAC) infectious clones. Through systematic investigation of the cleavage site positioning relative to the homologous arms, the research team significantly enhanced homologous recombination efficiency. This methodological advancement enabled rapid and efficient construction of pseudorabies virus BAC clones, representing a substantial improvement over conventional approaches [103]. To date, successful BAC-based infectious clones have been developed for over ten animal herpesviruses, including feline herpesvirus [104–106] and pseudorabies virus [83, 107], as well as for all human herpesviruses except Human herpesvirus 7 [96, 108–110].

Coronaviruses are the largest single-stranded RNA viruses, presenting formidable challenges in full-genome assembly due to their expansive genome sizes, the toxicity of specific genomic regions, the presence of mutations and deletions in the genomic sequence [111]. Prior to the development of full-length infectious clones, an initial reverse genetics system for coronaviruses was established, using the high efficiency of homologous recombination inherent to these viruses. This system was based on a targeted RNA recombination strategy [112, 113]. However, this approach is constrained by its dependence on the requirements for viral replication and propagation, which complicates the manipulation of replication enzyme genes and the investigation of lethal mutations [22]. In the year 2000, Almazan et al. first employed BAC for the construction of an infectious clone of the transmissible gastroenteritis virus (TGEV), which marked the inception of BAC-based infectious clones for the coronavirus family [99]. The research team assembled the complete cDNA of the TGEV genome into the BAC vector pBeloBAC11. This assembly was designed with the inclusion of a CMV immediate-early promoter at the 5'terminus, a 24-nucleotide polyA tail at the 3'terminus, and the sequences of the hepatitis delta virus ribozyme (HDVR) and bovine growth hormone (BGH) terminator. This innovative design ensured the encapsidation of viral RNA and facilitated the generation of infectious virus directly from the cDNA clone, thereby eliminating the requirement for in vitro transcription. Expanding upon the foundational work with BAC technology, the research group advanced the field by creating an infectious clone of the severe acute respiratory syndrome coronavirus (SARS-CoV) in 2006 [114]. Recent scholarly endeavors have showcased the successful amalgamation of BAC technology with conventional molecular biology techniques, utilizing unique restriction endonucleases to seamlessly integrate multiple genomic DNA fragments of SARS-CoV-2 into a BAC plasmid vector. The resultant rescued virus displayed growth kinetics and plaque morphologies that closely mirrored those of authentic SARS-CoV-2 isolates in cellular models [115, 116] Furthermore, BAC technology has been instrumental in the in vitro synthesis of full-length viral RNA using a T7 promoter, which, post-transfection, enables the generation of recombinant SARS-CoV- 2 [117]. This approach elegantly bypasses the necessity for nuclear transcription of infectious clones driven by the CMV promoter, thus circumventing potential complications associated with intron splicing. Beyond the realm of SARS-CoV-2 and TGEV, BAC vectors have been adeptly applied in the construction of infectious clones for a spectrum of other coronaviruses, encompassing Human Coronavirus OC43 (HCoV-OC43) [3], Middle East respiratory syndrome coronavirus (MERS-CoV) [22], swine acute diarrhea syndrome coronavirus (SADS-CoV) [118], Porcine delta coronavirus (PDCoV) [119] and feline infectious peritonitis coronavirus (FIPV) [18].

## Combined YAC-BAC recombination technology

In most cases, YAC and BAC have been used as independent technologies for the construction of infectious viral clones. With the development of yeast-bacterial shuttle vectors, researchers have integrated the highly efficient homologous recombination mechanism of yeast with the replication and manipulation advantages of *E.coli*, leading to the development of the YAC-BAC combined approach. This hybrid approach leverages yeast’s efficient homologous recombination for precise assembly of large constructs, which are then transferred to BACs for propagation and modification. The combined technology is particularly useful for studying complex genomes, constructing genomic libraries, and engineering large viral genomes.

### Combined YAC-BAC systems

When compared the use of YAC and BAC technologies for the construction of infectious clones, several elements take into consideration, including the key elements, plasmids, advantages, and disadvantages of each approach (Table 1**)**. For example, YACs can maintain and propagate larger genomic DNA fragments (up to 2 Mb) in yeast cells, while BACs can accommodate slightly smaller fragments (up to 300 Kb) in bacterial hosts. Furthermore, YACs contain the essential yeast centromere, telomeres, and autonomously replicating sequence, while BACs contain the F-factor origin of replication and partitioning elements. In summary, YACs offer the ability to maintain and study larger genomic DNA fragments in a more native context, while BACs are easier to manipulate and engineer using standard molecular biology techniques.

**Table 1:** Comparison of YAC and BAC Technology

**Technology**	**Main Elements and Function**		**Plasmid**	**Advantages**	**Disadvantages**
YAC	YAC cassette	Regulating the expression, isolation, and selection of target genes in yeast	pRML [51] pCC1BAC-his3 [1]	A large amount of DNA can be cloned. Applicable to any region of the viral genome. TAR fosters one-step genome assembly.	Chimeric, missing, and rearranged phenomena. Poor stability and low conversion efficiency
	ARS-like sequences	The origin of yeast replication			
	Hook	Connection between vector and target fragment			
	Selectable marker	Ensure that only cells that have been successfully transformed are retained			
BAC	ori	Stable replication of vectors in host cells	pBAC108L [78], pBeloBAC11 [79], pCC1BAC [89], pSMART BAC [92],	Adapt to large sequences without any risk of rearrangement. High Conversion efficiency; Easy to handle and operate	High AT content and sequences unsuitable for cloning. Transposition phenomenon of bacterial chromosomes
	repE	Encoding ATP driven helicase, responsible for initiating and maintaining DNA replication			
	ParA, parB and parC	Working together to control the copy number of BAC vectors			
	Multiclonial Site (MCS)	Inserting exogenous DNA fragments, usually located in the center of the vector, facilitates subsequent cloning operations			

*YAC* Yeast Artificial Chromosome, *BAC *Bacterial Artificial Chromosome, *ARS-like sequences like* Autonomous Replication Sequence, *Ori *Origin of Replication, *repE* Replication protein E, *ParA, parB and parC *Partitioning protein A, Partitioning protein B, Partitioning protein C

The development and application of YAC and BAC technologies has facilitated the isolation of large genomic fragments. The choice between YAC and BAC technologies for constructing infectious clones depends on the specific requirements of the project, such as the size of the viral genome, the need for maintaining native genomic context, and the available resources and expertise. However, the process of isolating target genes needs subcloning procedures involving thousands of YAC or BAC clones. Furthermore, when multiple genomic modifications are required, each modification must be done sequentially. This sequential approach considerably increases the time required for the construction of infectious clones.

The YAC-BAC approach, based on the principles of TAR, highlights the significance of BAC and presents a novel approach for constructing infectious virus clones. This technique primarily used the reverse genetics rescue system of RNA polymerase II. By incorporating the CMV promoter at the 5'terminus, this method eliminates the need for in vitro transcription or the use of T7 polymerase-expressing cell lines, which are required with the prokaryotic T7 promoter. Consequently, recombinant plasmids can be directly transfected into mammalian cells for in vivo transcriptional rescue of the virus. The viral genome is pre-designed into multiple overlapping fragments, each containing homologous repeat sequences at both ends, and subsequently linked to a YAC-BAC shuttle vector. The fragments and vectors are then introduced into yeast cells through transformation using polyethylene glycol (PEG)/lithium acetate (LiAC) and dimethyl sulfoxide (DMSO). The high efficiency of yeast in DNA uptake and homologous recombination facilitates the assembly and integration of these fragments. Selection is achieved through markers on the YAC-BAC shuttle vector; yeast cells lacking the viral genome or containing only the viral genome are unable to synthesize the histidine required for their growth, thereby failing to form colonies. In contrast, only those shuttle vectors that successfully incorporated the viral genome fragments possess the ability to produce histidine, resulting in the formation of yeast colonies. PCR analysis at the fragment junctions provides preliminary validation of yeast clones. Yeast colonies confirmed to be accurate and complete are selected for the extraction of recombinant yeast plasmids. These plasmids are subsequently introduced into *E.coil* through electroporation for plasmid amplification. *E.coli* colonies that test positive by PCR are then processed to extract plasmids, which, following sequencing, can be used for viral rescue applications. The YAC-BAC approach has been effectively employed for RNA viruses, including coronaviruses and flaviviruses, as well as for large DNA viruses such as herpesviruses.

Various YAC-BAC shuttle vectors have been developed for assembling infectious viral clones. Gibson refined the pCC1BAC vector by integrating yeast centromeric (*CEN6*) sequences, Autonomous Replication Sequence, and selectable yeast markers (*His3*), resulting in the creation of the pCC1BAC-His3 vector [120]. This enhanced vector has been successfully applied in research on various viral pathogens, including SARS-CoV-2 [1], Human simplex virus 1 **(**HSV- 1) [24], and feline infectious peritonitis coronavirus (FIPV) [9]. Additionally, researchers at the Wuhan Institute of Virology have developed the plasmid Genome Fast (pGF). shuttle vector, which significantly improves the assembly efficiency of complete viral cDNA [15, 121]. Furthermore, the commercially available pYES1L vector, with a capacity of up to 110 kb, has been widely applied in the reverse genetics of coronaviruses such as porcine epidemic diarrhea virus (PEDV) and HCoV-OC43 [29].

The YAC-BAC method integrates the merits of TAR technology, reduces the need for in vitro DNA manipulation, and eliminates the dependence on restriction endonucleases. This technique capitalizes on the high cloning efficiency of yeast and the minimal toxic impact on bacteria to produce recombinant yeast plasmids harboring full-length viral genomes. These recombinant plasmids, which may present at low concentrations and potentially contain yeast chromosomal contaminants, undergo further purification and amplification in *E. coli*. The purified plasmids are then transfected into mammalian cells to facilitate virus rescue.

When deploying this technology for constructing infectious clones, several factors should be considered. First, the selection of *E. coli* competent cells is crucial, as it can affect the rate of successful transformations and influence gene insertion, transposition, and deletion events. Furthermore, precise regulation of culture temperatures for both yeast and *E. coli* is imperative to maintain the efficiency of yeast recombination and prevent the induction of mutations in *E. coli*. Lastly, although PCR analysis of fragment junctions provides preliminary verification, sequencing of the finalized YAC-BAC constructs is indispensable to ensure genomic sequence accuracy and identify potential sequence anomalies.

### Application of YAC-BAC in complex viral genomes

The YAC-BAC approach combines the effective homologous recombination capabilities and low bacterial toxicity of YAC vectors with the stability and high transformation efficiency characteristics of BAC vectors. This integrated method circumvents the complex and expensive steps involved in yeast plasmid extraction and addresses the toxicity associated with partial viral sequences in *E. coli*. It uses a more straightforward and stable mRNA transfection method for viral rescue, eliminating the need for restriction endonucleases [122]. To date, infectious clones of multiple viruses have been constructed using YAC-BAC technology (Table 2**).**

**Table 2:** Examples of the Application of YAC-BAC Approach in the Construction of Infectious Clones for Different Viral Species

Family	Host	Genus	Species	Gene (kb)	Template	Assembly Strategy	Vector	*S.cerevisiae*	*E.coli*	Reporter gene	Growth kinetics	Viral Titer/Virulence
*Flaviviridae*	Human	Flavivirus	DEN2 NGC [123]	11	Passaging adaptation strain	Two steps	pRML	YPH857	Stabl2	/	Similar kinetics to parental virus	Titer is comparable with wild type
*Coronavirida*e	Human	Betacoronavirus	MERS-CoV [23]	31	Chemical synthesi**s**	Two steps	pYES1L	MAV203	DH10B	/	Similar kinetics to parental virus	/
	Human	Betacoronavirus	HCoV-OC43 strain VR 759 [29]	32	Chemical synthesis	Three steps	pEASY-Blunt pYES1L	MAV203	TOP10	GFP	/	First generation is low and titer increase after the third generation
	Feline	Alphacoronavirus	FCoV [9]	29-32	Plasmid	One step	pCC1BAC-His3	VL6-48N	EPI300	/	Similar kinetics to parental virus	Titer is comparable with wild type
	Porcine	Alphacoronavirus	PEDV strain HM [122]	29-32	Passaging adaptation strain	One step	pYES1L	MAV203	DH10B	/	Similar kinetics to parental virus	Titer is comparable with wild type
										EGFP	Reduced growth compared with wild type	Titer is lower than wild type and rPEDV
*Herpesviridae*	Human	Simplexvirus	HSV-1 strain KOS [124]	152	Virus, Plasmid	Two steps	pCC1BAC-his3	VL6-48N	DH10B TOP10	eBFP2/Cerulean/ mCherry/mNeptune2	Reduced growth compared with wild type	Titer is lower than wild type
	Human	Cytomegalovirus	HCMV strain Toledo [20]	235	Passaging adaptation strain	Three steps	pCC1BAC ura3 pCC1BAC-his3	VL6-48N	EPI300	/	Similar trends with wild type in MRC-5	Clear difference in Toledo-p in mice
	Human	Simplexvirus	HSV-1 strain H129 [125]	163	Plasmid	Three steps	pGF	VL6-48	EPI300	EGFP	Recombinant has more optimized growth properties than parental virus	Titer is higher than parental virus
	Human	Rhadinovirus	KSHV or HHV-8 [10]	138	Passaging adaptation strain	One step	pCC1BAC-his3	VL6-48	DH10B	GFP	/	Titer is comparable with wild type
	Mouse	Cytomegalovirus	RCMV [10]	203	Clinical strains	One step	pCC1BAC-his3	VL6-48	DH10B	/	Similar kinetics to parental virus	Titer is comparable with wild type
*Baculoviridae*	Arthropod	Alphabaculovirus	AcMNPV [15]	145	Virus	Four steps	pGF	VL6−48N	EPI300	EGFP	Similar kinetics to parental virus	Viral titer and virulence are comparable with wild type

*DEN2 NGC *Dengue virus type 2, *MERS-CoV *Middle East Respiratory Syndrome coronavirus, *FCoV *Feline coronaviruses, *PEDV strain HM *Porcine epidemic diarrhea virus strain HM, *HSV-1 strain KOS *Herpes simplex virus type 1 strain KOS, *HCMV strain Toledo *Human cytomegalovirus strain Toledo, *HSV-1 strain H129 *Herpes simplex virus type 1 strain 129 (H129), *KSHV or HHV-8*Kaposi's sarcoma-associated herpesvirus or Human herpesvirus 8, *RCMV*Rat cytomegalovirus, *AcMNPV Autographa californica *nucleopolyhedrovirus, *GFP *Green fluorescence protein, *eBFP2*enhanced Blue Fluorescent Protein 2, *Cerulean *Enhanced Cyan Fluorescent Protein, *mCherry*monomeric Cherry fluorescent protein, *mNeptune2*monomeric Neptune fluorescent protein 2, *EGFP*enhanced green fluorescence protein

Polo et al. first demonstrated the application of YAC-BAC approach in flavivirus research as early as 1997 by constructing an infectious clone of Dengue virus type 2 (DEN2 NGC). The DEN2 genome was segmented into four parts, which were reassembled into a complete infectious clone through a two-step assembly process [123]. First, three segments of the viral genome were ligated to a linearized shuttle vector, followed by replication and screening in *E. coli*. The correctly assembled segments were then recombined with RT-PCR amplified fragments in yeast to produce yeast clones carrying the full-length genome. The yeast plasmids were subsequently transfected into *E. coli* for further amplification and purification. Positive clones were validated by colony PCR and restriction enzyme digestion before being transfected into mammalian cells for viral rescue. The attempts to construct infectious clones directly in *E. coli* were unsuccessful due to cell toxicity issues. Concurrently, YAC experiments demonstrated that among 26 yeast clones screened, 24 had incorrect restriction patterns, indicating a low yield and cumbersome procedure with additional steps. In contrast, the YAC-BAC approach achieved a higher accuracy rate, with 5 out of 6 yeast clones containing the expected full-length DEN2 cDNA, and *E. coli* results showing an accuracy rate of 87.5%. This suggests that the YAC-BAC approach provides enhanced accuracy and efficiency in constructing infectious clones.

In 2016, Nikiforuk et al. employed the YAC-BAC approach for human coronaviruses, using a two-step assembly strategy to create an infectious clone of Middle East Respiratory Syndrome coronavirus (MERS-CoV) [23]. The researchers constructed overlapping fragments of the chemically synthesized viral genome, which were interconnected by 30 bp repetitive sequences. The 5'end of the viral genome was designed to include a CMV promoter, while the 3'end incorporated a β-globin terminator and a hepatitis delta virus ribozyme to facilitate in vivo transcription. Homologous recombination was conducted in yeast cells, and the resulting recombinant plasmids were amplified and purified in *E. coli.* Colony PCR analysis revealed an overall efficiency of approximately 95% for yeast transformation, homologous recombination, and screening processes. The sequencing confirmed that the rescued virus was identical to the clinical isolate MERS-CoV/EMC-2012 cDNA. Correctly identified plasmids were subsequently transfected into BHK-21 cells for virus rescue. After three blind passages in Vero cells, infectious virus particles were detected in the supernatant. RT-PCR was employed to amplify and sequence the extracted viral RNA, verifying successful virus rescue. Furthermore, replication kinetics analysis demonstrated that the recombinant virus rMERS-CoV had replication dynamics comparable to those of the clinical isolate MERS-CoV EMC-2012, confirming that the recombinant virus is a reliable phenotypic substitute for the clinical isolate.

Using the YAC-BAC approach, Tan et al. developed a streamlined method for constructing infectious clones of the HCoV-OC43 VR1558 strain. This approach demonstrates the efficacy of the YAC-BAC reverse genetics system in replicating all functions of the *wild-type* virus, thereby eliminating the need for complex cloning procedures and enabling clone construction within one week. The genome of HCoV-OC43 VR1558 was segmented into eight overlapping fragments, which were inserted into the YAC-BAC shuttle plasmid pYES1L. Positive clones were generated through homologous recombination in *Saccharomyces cerevisiae*, followed by transformation into *E. coli* to produce infectious clone plasmids. The study explored insertion, partial replacement, and complete replacement strategies within the ns2 region, resulting in three distinct infectious clones, including *wild-type* strain, Enhanced Green Fluorescent Protein (EGFP) reporter recombinant, and Renilla Luciferase (RLUC) reporter recombinant. Detailed analyses of replication dynamics, cytopathic effects, antigen expression, and genetic stability of the VR1558 strain in BHK-21 cells were conducted. Additionally, lethality, weight variation, viral load in various tissues, and in vivo imaging in BALB/c mice were evaluated. A high-throughput visual platform was subsequently established for drug evaluation, which screened and validated the antiviral efficacy of four drugs [126]. Another research group modified the Open Reading Frame 5 (ORF5) region of the HCoV-OC43 mouse-adapted strain and applied a three-step construction strategy to generate infectious clone of HCoV-OC43 strain of VR759 with a green fluorescent reporter gene [29].

Furthermore, the application of YAC-BAC technology has been extended to the study of animal coronaviruses. Zhou et al. constructed infectious clones of the GII-type PEDV with a streamlined, one-step construction strategy [122]. The expression of the virus-specific nucleocapsid protein was confirmed through indirect immunofluorescence assays and western blot analysis, while the presence of engineered silent mutations was validated by RT-PCR and sequencing. The biological characteristics of the recombinant virus were evaluated by multi-step growth curve analysis and plaque assays, indicating it had similar traits to the parental virus. In addition, researchers employed the CRISPR/Cas9 gene editing system to construct PEDV reporter viruses. Comparative analyses revealed that recombinant viruses with EGFP reporter genes and T2 A self-cleaving peptides inserted at the N-terminus of the nucleoprotein displayed elevated levels of fluorescence expression relative to that of inserted within the ORF3 region. These comparative studies helped researchers to evaluate the influence of gene position on the performance of reporter viruses, such as the intensity of reporter gene expression, the capacity for viral replication, and the impact on host cellular processes. The research was helpful for refining the design of reporter viruses, enhancing the sensitivity and precision of experimental outcomes.

In 2023, Cao et al. used YAC-BAC approach to construct four different types of feline coronavirus (FCoV) [9]. By replacing the S gene of the highly pathogenic type II feline infectious peritonitis virus of FIPV DF-2, which is adapted to cells, with the S genes of the low-virulence FIPV and the highly infectious feline enteric coronavirus (FECV) I MG89351, which cannot be cultured in cells, they obtained recombinant viruses with similar growth characteristics to the FCoV DF-2 strain. The results indicated that the S gene could not induce the transformation of non-virulent viruses into pathogenic ones, providing a powerful tool for studying FCoV transmission, pathogenesis, and evaluating for Feline Infectious Peritonitis vaccines and treatments [9]. Furthermore, the researchers conducted a comparative analysis of the impact of different promoters on the construction of infectious FCoV clones using YAC-BAC approach. The study revealed that both the T7 and CMV promoters were equally effective in rescuing recombinant viruses, with no statistically significant differences in viral titters observed. Notably, the use of the CMV promoter eliminates the requirement for in vitro transcription or the use of a T7 polymerase-expressing cell line, thereby streamlining and expediting the viral rescue process, which is a significant advancement in the field of molecular virology.

The YAC-BAC approach has proven effective across a spectrum of viral research areas, extending its utility to both RNA and large DNA viruses. In 2017, Oldfield et al. constructed an infectious clone of the HSV-1 KOS strain with a 152 kb genome [24]. They assembled the complete viral genome in yeast competent cells VL6-48 N using 80 bp overlapping sequences fragments. The recombinant plasmid containing the complete genome was electroporated into the *E. coli* EPI300, and positive clone plasmids were transfected into mammalian cells to rescue the recombinant virus. Furthermore, the utility of this modular assembly technique was demonstrated by modifying individual genes multiple times, altering two genes simultaneously, and performing single and combined deletions of five conserved genes encoding viral particle structural proteins. Furthermore, Knickmann et al. employed a single-step YAC-BAC method to rescue rat cytomegalovirus (RCMV) and Kaposi's sarcoma-associated herpesvirus (KSHV/HHV-8), demonstration the versatility of the YAC-BAC method in herpesvirus studies [10].

The YAC-BAC platform has also been employed in baculovirus reverse genetics research. In 2017, Shang et al. successfully synthesized the genome sequence of the baculovirus Autographa californica nucleopolyhedrovirus (AcMNPV), excluding the hr4a region, using a multi-step assembly process. The virus, AcMNPV-WIV-Syn1, was subsequently rescued from Sf9 insect cells [15]. An infectious clone of AcMNPV-WIV-Syn1, incorporating the reporter gene EGFP, was also constructed [127]. This innovation serves as a crucial tool for detailed investigations into the biological characteristics of baculoviruses.

### Future of YAC-BAC technology

The YAC-BAC approach, which combines the strengths of yeast artificial chromosome and bacterial artificial chromosome technologies, provides a versatile platform for the rapid and adaptable acquisition of viruses. This method is particularly useful for studying large and dangerous viruses, enhancing our understanding of viral genome complexities, pathogenicity, vaccine development, and antiviral strategies. However, despite its considerable advantages, the method has limitations and needs further improvement. Its applicability is currently focused on large DNA viruses and single-stranded RNA viruses with positive sense, with limited attention on negative-sense RNA viruses. Construction of infectious clones using this method requires hosts from different kingdoms—yeast and *E. coli*—which may introduce genomic aberrations. The efficiency of transformation and the stability of viral constructs can vary depending on the host, potentially affecting reproducibility and the feasibility of specific experiments. Moreover, the complexities associated with YAC and BAC technologies may hinder their broader adoption and operational efficiency, thereby limiting research throughput and scalability. These constraints could impede the overall progression of research and the reproducibility of experimental results. Therefore, while the method offers significant advancements, the identified limitations highlight the need for ongoing refinement and careful addressing of practical and safety challenges.

## Applications for viral infectious clones in virology

**Fig. 2:**
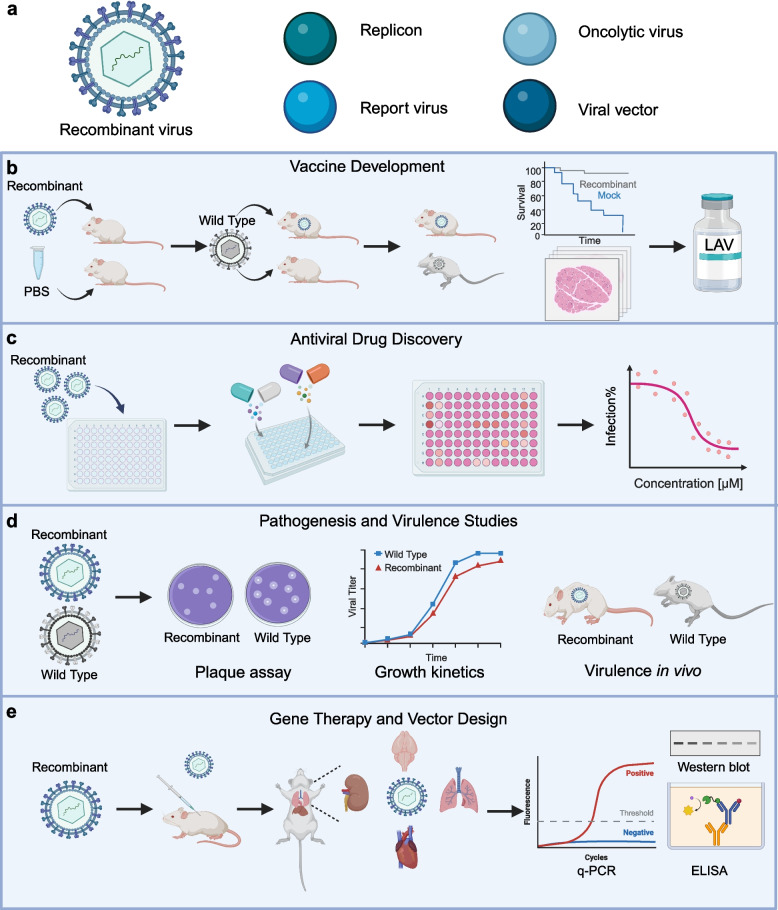
Overview of recombinant viruses generated by infectious cloning and their applications in biomedical research, such as vaccine development, antiviral drug discovery, pathogenesis and virulence studies, gene therapy and vector design**. a** Examples for recombinant virus models in virology research, including replicons, oncolytic viruses, reporter gene viruses, and viral vectors. **b** The application of infectious cloning in vaccine development. Recombinant attenuated viruses constructed by infectious cloning can protect the challenge of wild type viruses in animal models. For example, in a mouse model, those immunized with a recombinant attenuated virus survived from *wild-type* virus challenge, while the control mice immunized with PBS did not. Survival curve and pathological results indicate that the recombinant virus could serve as a candidate attenuated live vaccine. **c** Recombinant viruses can facilitate antiviral drugs discovery. By culturing recombinant virus with candidate antiviral drugs, researchers can evaluate the efficacy of these drugs. The use of recombinant virus with specific advantages (e.g., reporter genes, attenuation), allows for the statistically determination of the half-maximum inhibitory concentration of the drugs**. d** The recombinant viruses for viral pathogenesis and virulence. By comparing the recombinant virus with specific mutation to their wild-type viruses, researchers can evaluate differences in growth characteristics and virulence in vitro and in vivo. **e** Recombinant viruses for gene therapy and vector design. The virus recombinant virus can be used in vivo, allowing for the analysis of various tissue samples and detection of therapeutic effects

The use of YAC, BAC, and YAC-BAC technologies has rescued recombinant viruses including replicons, oncolytic viruses, reporter gene viruses, and viral vectors. These viruses have been widely used in various virology related fields, including vaccine development, antiviral drug discovery, pathogen and virulence research, as well as for gene therapy and vector design (Fig. 2). Many of these experiments involve in vivo animal studies, providing important tools for evaluating the immunogenicity, safety, and efficacy of vaccines, as well as for further development and optimization of oncolytic viruses, and basic research in virology (Table 3).

**Table 3:** Application of YAC or BAC approach for viral infectious clones in preclinical animal experiments

Application	Virus			Animal		Pathway
	Parental Virus	Recombinant virus	Purpose	Animal models	Old	
Vaccine Development	MERS-CoV	dORF3-5 MERS-CoV [128]	The impact of ORF3-5 on the pathogenesis	CRISPER-generated 288-330^+/+^C57BL/6 mice	20 weeks	intranasally inoculated
	SARS-CoV-2	double ORF-deficient rSARS-CoV-2 [149]	The impact of dual ORF deletion on viral replication	K18 hACE2 transgenic mice	5 weeks	intranasally inoculated
				golden Syrian hamsters		
	HCMV	Toledo-P [20]	Study the latent period and in vivo reactivation ability of clinical isolates	NOD-scid IL2R_c_^null^(NGS)mice	>12 week	intraperitoneal injection
	ZIKV	ZIKV cDNA clone [129]	The feasibility of rescuing viruses in vivo	IFNAR^−/−^A129 mice	4-5 week	inoculated in the footpad in the quadriceps muscle in the peritoneal cavity
Pathogenesis and Virulence Studies	TGEV	TGEV-GFP-ΔS_NTD [130]	The effect of S-NTD224 TGEV on the virulence of TGEV	piglets	2 days	intranasally and orally inoculated
	FCoV	rQS-79 [131]	Constructing an in vivo model for studying the pathogenic mechanism of FIPV	cats	1-2 year	orally inoculated
	ZIKV	rZIKV-RGN[132]	The effect of NS2A protein residues on viral RNA synthesis and pathogenesis in vivo	type-I interferon (IFN) receptor deficient (IFNR-/-) A129 mice	4-6 week	inoculated in the footpad
Gene Therapy	Measles virus	rMV-Hu191 [133]	In vivo toxicity changes of rMV-Hu191 mutant with SAM binding site	cotton rats (Envigo)	4-6 week	intranasally inoculated
	rMeV Hu191 [134]	/	To study the multiple anti-tumor effects of rMeV Hu191 on breast cancer	Nude mice	/	inject into tumor
Vector Design	HAdV-3	rHAdV-3[217]	Study on the expression of rHAdV-3 specific antigen	BALB/c	4-6 week	intranasal route intramuscular route

*MERS-CoV *Middle East Respiratory Syndrome coronavirus, *SARS-CoV-2 *Severe Acute Respiratory Syndrome Coronavirus 2, *HCMV *Human cytomegalovirus, *ZIKV *Zika Virus, *TGEV *Transmissible Gastroenteritis Virus, *FCoV *Feline coronaviruses, *rMeV Hu191 *recombinant measles virus vaccine strain Hu191, *HAdV-3* Adenovirus Type 3

### Vaccine development

Several candidate vaccines have been developed, including live attenuated vaccines (LAVs), inactivated vaccines, viral vector vaccines, protein subunit vaccines, DNA and mRNA vaccines, and virus-like particle (VLP) vaccines [135]. LAVs reduced the pathogenicity of the microorganism, simulating the natural infection process to induce both cellular and humoral immune responses, thereby providing sustained immune protection. Traditional attenuation methods typically rely on random mutations or long-term adaptive cultivation [136–138], whereas reverse genetics modifies genomes by targeted mutagenesis to precisely alter genes associated with pathogenicity, thus attenuating the virus [139–141] and enhancing vaccine stability [142–144]. This approach significantly shortens the preparation time for live attenuated vaccines, enabling the rapid design of vaccine candidates and providing a powerful tool for the development of LAVs [145–148].

Vashee et al. use the TAR method to assemble clinical isolation Human Cytomegalovirus (HCMV) Toledo-P. The Toledo-P strain displays characteristics of primary isolates, including broad cellular tropism in vitro and the ability to set latency and reactivation in humanized mice. Modified TAR fragments retained the ability of HCMV to elicit lasting effector memory, while incorporating safety features that reduced viral pathogenesis. These findings offer new insights into vaccines and vectors derived from HCMV Toledo [20].

Mutations in viral innate immune-related non-structural proteins can enhance the safety and stability of live attenuated vaccines, as some viral proteins are critical for viral replication. Menachery et al. demonstrated that the deletion of non-structural protein ORF5 in MERS-CoV activates the host interferon pathway, enhancing NF-κB activation and the inflammatory response. In vivo experiments show that the recombinant virus with ORF5 deletion had replication attenuation early in infection, but generates a protective immune response comparable to the wild-type virus [128], suggesting the potential of ORF5 deletion as an attenuated live vaccine. Ye et al. employed BAC technology to simultaneously delete two auxiliary proteins (ORF3a and ORF7b) in SARS-CoV-2, creating three dual-ORF-deficient recombinant SARS-CoV-2 viruses. All the three recombinant viruses induced strong innate and adaptive immune responses in K18 hACE2 transgenic mice, and the recombinant with ORF3a/ORF7b deletion and S protein modification, combines virulence attenuation with immunogenicity and vaccine update feasibility, making it a safe and protective LAV candidate for preventing SARS-CoV-2 infection and associated COVID-19 disease [149].

Traditional reverse genetics techniques often rescue viruses using cell models and conduct in vivo infectious studies after prolonged viral passage. This process not only delays the identification of the virus biological characteristics in vivo but also may lead to changes in the virus phenotype due to mutations during the cell passage process [150]. In contrast, in vivo reverse genetics directly rescues viruses from validated animal models, which not only accelerates the related research on recombinant viruses but also simplifies the development of LAV vaccines based on these attenuated virus forms. Luis Martínez-Sobrido et al. rescued the infectious cDNA clone of the ZIKV in IFNAR^−/−^ A129 mice, which lack a type I interferon response [129]. The rescued virus effectively replicates in vivo, inducing a strong humoral immune response, including high levels of neutralizing antibodies, and providing ZIKV protection. The strategy of rescuing infectious cDNA clones in vivo combines the advantages of broad innate and adaptive immune responses generated by LAV vaccines with the stability, ease of production, and reduced cold-chain requirements of DNA vaccines, significantly improving vaccination strategies for a variety of pathogens.

### Antiviral drugs discovery

Viral infections cause various infectious diseases, posing a significant threat to human health. Despite recent advances in drug development, effective treatments for most viral infections remain lacking [151, 152]. Antiviral drugs can provide additional protection when vaccines are difficult to disseminate or prove ineffective, thus complementing vaccination efforts. Traditional antiviral drug screening methods, based on viral antigens and/or plaques, with limitations such as low throughput and biosafety concerns and hinder large-scale testing and restrict the discovery of new drugs [153, 154]. However, the application of reverse genetics techniques has made reporter genes and replicons valuable tools for high-throughput screening of antiviral drugs [153, 155].

Reporter genes have been widely applied in virological research as indicators of gene expression changes and have been used in the study of various viruses, including influenza virus [156], dengue virus [157], vaccinia virus [158], SARS-CoV-2 [159–161] and Hepatitis C virus [162, 163]. Fluorescent reporter genes can be divided into two categories: luciferin and luciferase. The former, as an autocatalytic fluorescent protein, enables direct imaging and dominates in experiments involving microscopy [164]. In contrast, luciferase catalyzes the substrate to produce spontaneous fluorescence through an enzymatic reaction. Bioluminescence imaging has the lowest phototoxicity and is suitable for in vivo imaging of mammalian subjects. Recombinant viruses expressing reporter genes overcome the limitations of auxiliary methods for detecting viruses in infected cells, providing important technological support for the rapid visualization evaluation of vaccines and drugs [160].

The COVID-19 pandemic, caused by SARS-CoV-2 infection, is characterized by rapid transmission and the continuous emergence of viral variants. Although vaccines are generally effective in preventing SARS-CoV-2 infection or mitigating the severity of COVID-19, their efficacy is influenced by the emergence of new viral variants and the decline in immunity over time [165]. Promising antiviral drugs, such as Paxlovid and Molnupiravir have demonstrated potential in treating COVID-19 and alleviating long-term symptoms associated with the disease. However, issues of viral rebound and drug resistance have been observed [166–168]. Viral variability and drug resistance indicate the urgent need to accelerate the development of antiviral drugs. As SARS-CoV-2 is classified as a Biosafety Level 3 (BSL-3) pathogen, research and the development of effective antiviral drugs face certain barriers.

The viral replicon, due to its lack of structural proteins and inability to produce infectious viral particles, has become a safe tool for the study of high-pathogenic viral replication and antiviral drug screening [81]. The replicon can complete only a single round of infection in the absence of heterologous protein expression, making it safer and allowing for viral research to be conducted without BSL-3 containment requirements. The spike protein (S) of SARS-CoV-2 primarily binds to the ACE-2 receptor on the surface of host cells, playing a critical role in viral infection. Researchers from Sun Yat-sen University used the BAC method to construct a fluorescent reporter gene replicon lacking the S protein. The results of drug evaluation with four known antiviral transcription/replication drugs of Remdesivir, Lopinavir, Ritonavir, and Carmofur, indicated that the luciferase-tagged replicon showed sensitivity comparable to that of the live virus [116]. Merck et al. constructed a replicon system targeting NSP-based antiviral drug activity, they replaced the spike protein with a fusion protein encoding luciferase and GFP, then substituted the sequence between the envelope protein (E) and membrane protein (M) with a neomycin resistance gene (Neo). Using this system, they developed a high-throughput drug screening system using this replicon and conducted pharmacodynamic evaluations of 27 drugs [169], their research also found efficacy differences for several SARS-CoV-2 drugs in different cell lines, highlighting the challenges of developing antiviral drugs that inhibit viral replication in vivo, and emphasizing the importance of drug evaluation in multiple cell lines. Researchers from China to construct a SARS-CoV-2-GFP replicon based on the YAC-BAC system, they replaced the spike protein genome with GFP-BlaR, deleted NSP1 and inserted a CMV promoter at the 5'end to drive viral genome transcription. The SARS-CoV-2-GFP replicon was used to evaluate the pharmacodynamics of the antiviral drugs E64-D and Remdesivir. GFP fluorescence intensity and Western blot results of tGFP-BlaR demonstrated that these drugs could inhibit the SARS-CoV-2-GFP replicon in a dose-dependent manner in 293 T cells. They also developed a GFP-Fluc dual-reporter gene replicon system, allowing different laboratories to select appropriate metrics for pharmacodynamic evaluations [170].

As the single-cycle replicon system requires transient transfection of vectors and RNA for each replication detection, making high-throughput screening and other research operations cumbersome [171]. Furthermore, transiently expressed RNA replicons have certain toxicity to cells, preventing sustained replication within the cells, leading to short replication detection times and unstable results in different cell lines [172]. To address these limitations, Tony Wang et al. reported a stable cell line with self-replicating SARS-CoV-2 RNA. They introduced K164 A/H165 A mutations into Nsp1 to reduce the virus's toxicity to cells and constructed the reporter gene replicon named SARS-CoV-2-Rep NanoLuc-Neo-Nsp1 K164 A/H165 A. This replicon transiently expresses the reporter gene after electroporation into BHK-21 cells stably transfected with a tetracycline-inducible N protein-expressing plasmid. Under G418 selection pressure, 12 surviving cell lines were obtained, and these cell lines stably express the reporter gene after 20 passages, with active replication capabilities of the replicon. The authors further evaluated the antiviral effects of 273 compounds targeting SARS-CoV-2 NSP5 (3 CLpro), NSP3 (PLpro), NSP12 (RdRP), NSP15, NSP16, and X region using the replication subsystem. Based on the expression of nano-luciferase, nine compounds at a concentration of 5 mM showed efficacy above 50% inhibition. Among these, three compounds—Darapladib, Genz-123346, and JNJ-5207852—demonstrated cell type-specific inhibitory activity and may serve as inhibitors of SARS-CoV-2 [173]. In the same year, researchers constructed a stable cell line, VeroE6/Rep3, using VeroE6 cells and found that the RNA-dependent RNA polymerase inhibitor Molnupiravir have stronger inhibitory effects in VeroE6/Rep3 cell line than in transiently transfected VeroE6 replicon cells, This development provide a powerful tool for high-through screening of SARS-CoV- 2 antiviral drugs [171].

### Pathogenesis and virulence studies

Infectious clones can be generated by artificially synthesizing or modifying the viral genome, allowing for the observation of the impact of genetic alterations on viral biological properties. This approach reveals the pathogenic and mutagenic mechanisms of the virus. The bifunctional nsp14 subunit of the coronavirus replicase contains both a 3’− 5’ exoribonuclease (ExoN) and a guanine-N7-methyltransferase (N7-MTase) domain. The ExoN exon domain possesses proofreading activity, which enhances the replication fidelity of large and medium-sized Nidoviruses [174, 175]. Ogando et al. used BAC to generate a set of ExoN exon-mutated MERS-CoV nsp14 recombinant viruses. Of the 13 ExoN exon domain knockout mutants, none were successfully rescued, with only one highly conserved mutant rescued and capable of replication. In vitro assays revealed that all lethal knockout mutants significantly reduced ExoN activity but did not affect N7-MTase activity. Furthermore, the team also performed knockout mutations on the ExoN gene of SARS-CoV-2 and found that it failed to be rescued. This was in contrast to previously reported SARS-CoV exon knockout mutant phenotypes, suggesting that the ExoN exon not only possesses proofreading activity that enhances replication fidelity [176, 177], but also plays a crucial role in primary viral RNA synthesis [178].

The spike protein is a major pathogenic factor of coronaviruses; however, recent studies indicate that its role may not be as critical as previously expected for α-coronaviruses [131]. Combined with BAC and CRISPR-Cas9 technologies, researchers constructed a recombinant virus with a deletion of 224 amino acids in the TGEV spike gene (S_NTD224), revealing this deletion significantly impacts the growth kinetics of TGEV in PK-15 cells. In vivo experiments demonstrated that, regardless of whether the S_NTD224 is present or deleted, the recombinant TGEV virus induces pronounced clinical symptoms and mortality, suggesting the S_NTD224 deletion mildly affects TGEV virulence but does not determine enter-tropism, providing new insights for the development of novel attenuated TGEV vaccines [130]. Wang et al. constructed a series of chimeric mutants with the highly virulent PEDV BJ2011 C and the avirulent CHM2013 strains, identifying the structural protein coding region and 3'UTR as the key determinants of virulence in high-virulence PEDV variants, while the S gene is only one factor contributing to virulence variation [179]. Studies on the FCoV S gene showed that recombinant viruses deleted 245 amino acids from the 5'end of the S gene in the FCoV strain C2663 retain virulence in cats [180].

Infectious clones also have been used to study the pathogenesis and virulence mechanisms of other RNA viruses. ZIKV, an emerged mosquito-borne member of the Flaviviridae family, caused a global public health emergency in 2016 [181]. Silvia Márquez-Jurado et al. used a BAC-based infectious clone to investigate the effects of a single amino acid mutation (A175 V) in the NS2 A protein on the replication and pathogenicity. The results demonstrated that A175 V mutation significantly inhibited RNA synthesis and viral production in vitro and attenuated the virus in A129 mice, furthermore, single dose of the mutant virus was sufficient to confer protection against the parental *wild-type* (*WT*) ZIKV [132]. Yuta Kanai et al. demonstrated that modifying of NSP2 and NSP5 enabled the rapid and efficient generation of recombinant rotavirus. Based on this approach, recombinant rotaviruses with low replication and carrying the NSP4 mutant lacking the double-layer particle domain were successfully rescued, confirming the critical role of amino acids 161–175 of NSP4 in its interaction with VP6, as NSP4 knockout disrupted the formation of VP6 DLP structures [182].

### Gene therapy and vector design

**Fig. 3:**
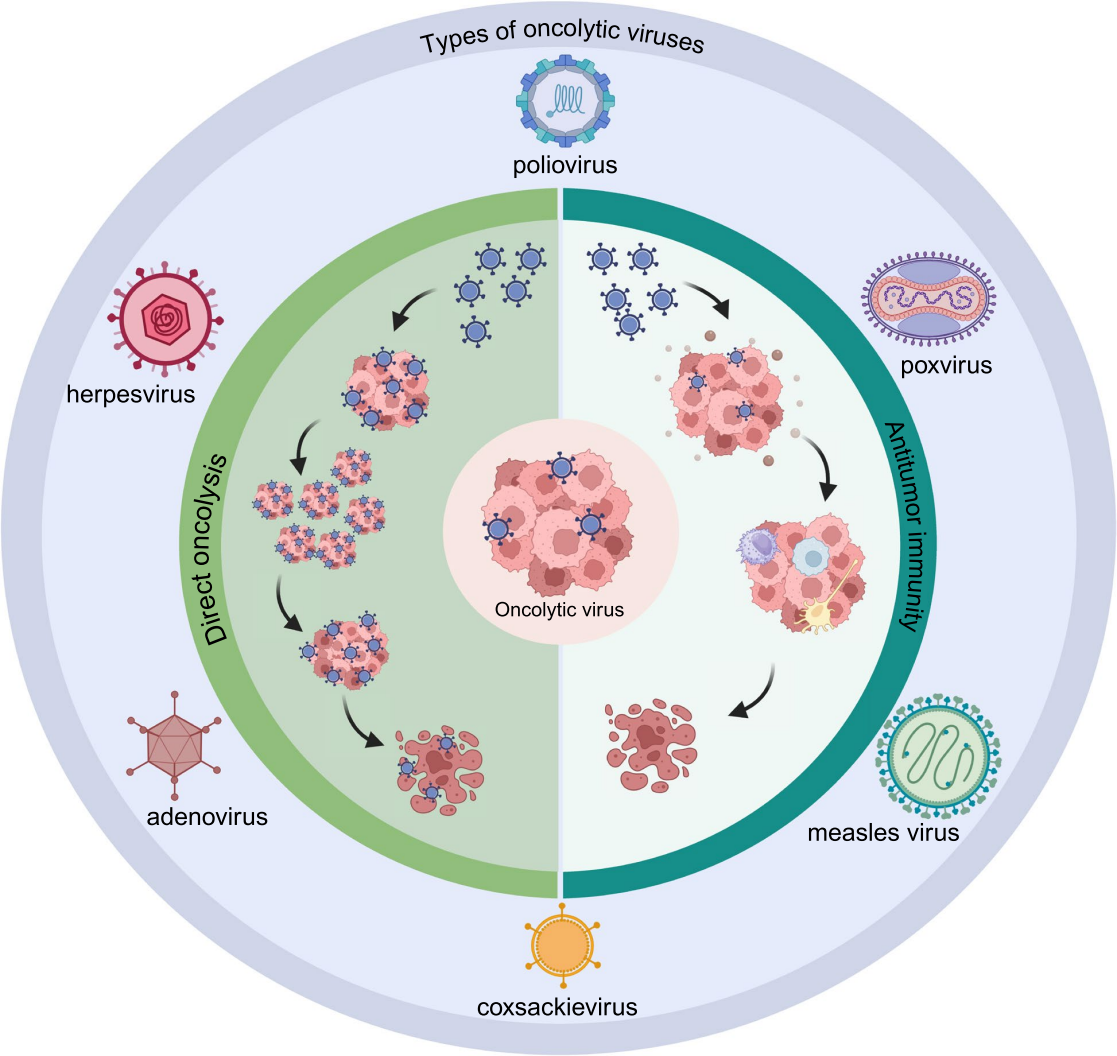
Overview of the types of oncolytic viruses and their mechanisms in targeting and destroying cancer cells. Oncolytic viruses that are commonly used in anti-tumor research including poliovirus, herpesvirus, poxvirus, adenovirus, measles virus, and coxsackievirus. There are mainly two aspects of oncolytic viruses for anti-tumor effect. One is direct oncolytic effect. Oncolytic viruses specifically replicate in tumor cells, it causes lysis of the tumor cells. The other is activation of antitumor immunity, immunogenic cell death induced by oncolytic virus exposure leads to the release of various molecules and recruitment of immune cells such as macrophages and dendritic cells, inducing the body to produce an immune response

Oncolytic virus immunotherapy is rapidly gaining interest in the field of cancer immunotherapy. The minimal toxicity, the direct oncolysis and immune activation make oncolytic viruses therapy become an promising treatment approach [183]. The advancements in genetic engineering and molecular virology allow for the customization of viruses with tumor specificity and safety. Oncolytic viruses are naturally occurring or genetically modified DNA and RNA viruses that selectively target tumors. Oncolytic viruses can be categorized based on their origin into naturally tumor-selective viruses and genetically engineered viruses. To date, various types of oncolytic viruses have been studied, including poliovirus, herpesvirus, poxvirus, adenovirus, measles virus, and coxsackievirus [184–187]. Anti-tumor effects are exerted through various mechanisms, including the direct lysis of tumor cells and the activation of anti-tumor immune responses [188, 189] (Fig. 3). And clinical applications of Oncolytic viruses in cancers such as glioma, melanoma, myeloma, and breast cancer have been widely evaluated [185, 190–192].

Herpes simplex virus type 1 (HSV-1) has a large genome, which allows it to incorporate a variety of transgenes, and it possesses high infectivity, broad host cell adaptability, and relatively safe genome editing capabilities, making it an ideal oncolytic virus [193]. Using reverse genetics technology to modify the genome of HSV-1, some have entered the clinical trial stage. G207 is the first oncolytic HSV-1 used in clinical trials in the United States, it was engineered by deleting the HSV-1 γ34.5 gene and inserting the *E.coli* *lacZ* gene into the UL39 (ICP6) gene, reducing its replication ability in health cells while maintaining effective replication in rapidly dividing tumor cells [194, 195]. This modification results in minimal toxicity in health cells while retaining sensitivity to antiviral drugs such as acyclovir, allowing for rapid suppression of the virus in case of adverse reactions [196]. Currently, G207 has been tested in several clinical trials, demonstrating its antitumor effects [197, 198]. Oncolytic virus G47∆ is a third-generation recombinant HSV-1 based on G207, further modified by deleting the α47 gene and introducing an overlapping US11 promoter, which enhances its replication in cancer cells and tumor specificity [199]. G47∆ has been shown to be effective in animal tumor models of various cancers, including brain tumors, prostate cancer, breast cancer, and neurofibromas, and has been approved in Japan as an oncolytic therapy for malignant gliomas [200–202]. Additionally, research has been conducted to modify the HSV- 1 genome to improve the safety and efficacy of oncolytic herpes simplex viruses (oHSVs). Inflammatory chemokines, such as C–C motif ligand 5 (CCL5), regulate immune cell trafficking in the tumor microenvironment and control tumor progression, making them promising targets for cancer therapy [203, 204]. However, the short half-life of CCL5 limits its effectiveness as a cancer therapy, and the introduction of oncolytic viruses can effectively address this issue. Yu et al. constructed the oncolytic virus OV-Cmab-CCL5 using bacterial artificial chromosomes, they inserted a bispecific fusion protein encoding the IgG1 form of an anti-epidermal growth factor receptor (EGFR) antibody, cetuximab, and cloned the CCL5 into a shuttle plasmid under the HSV pi4/5 promoter. The OV-Cmab-CCL5 was targeted to EGFR^+^ glioblastoma (GBM) tumor cells, significantly enhancing the migration and activation of natural killer cells, macrophages, and T cells. In mice bearing human and murine orthotopic GBM xenografts and syngeneic tumors, the"cold"tumors were converted into immunologically"hot"tumors, significantly prolonging survival and providing a promising approach for improving oncolytic virus (OV) therapy in solid tumors [205].

In addition, the measles virus has also been applied in oncolytic virus research. Zhao et al. constructed a recombinant vaccine strain named rMeV Hu191 with enhanced safety and immunogenicity [133], they replaced a single amino acid at the s-adenosylmethionine (SAM) binding site of the measles virus L protein. This team further investigated the efficacy of rMeV Hu191 against human breast cancer and its potential anti-tumor effects. The results indicated that rMeV Hu191 primarily enters BC cells via the CD40 receptor, inducing apoptosis, inhibiting cell proliferation, and promoting senescence, thereby exerting oncolytic effects. In a breast cancer xenograft mouse model, intratumorally injection of rMeV Hu191 significantly promoted tumor regression and prolonged mouse survival. The selective targeting and cytotoxicity of rMeV Hu191 against cancer cells highlights its clinical potential as a promising adjunct therapy for breast cancer treatment [134].

Advancements in synthetic biology have successfully modified various viruses to serve as viral vectors, including adenovirus [206], vaccinia virus [207, 208], and herpesvirus [209, 210]. For example, Adenovirus vectors are widely used in the development of candidate vaccines for infectious diseases due to their advantages, such as ease of genome manipulation, ability to accommodate large foreign gene inserts, high titers, and efficient transduction [211]. Adenovirus can induce innate immune responses and elicit adaptive immunity directed against both the vector and the transgene. After the adenovirus administration, antigen-presenting cells (APCs) transport the vector, vector proteins, or transgenes to the draining lymph nodes, where naïve T cells differentiate into CD4^+^ and CD8^+^ T cells. As MHC class I or II molecules recognize viral or transgene epitopes, both cellular and humoral immune responses are activated, ultimately resulting in the clearance of the vector and infected cells through neutralizing antibodies and CD8^+^ T cells [212–214]. Hexon is the major capsid protein in adenovirus and could induce highly efficient and persistent antibodies [215, 216]. Based on the commercially available HAdV-5 vector, researchers have constructed a recombinant attenuated human adenovirus vaccine carrying the complete gene for HAdV- 3 hexon using homologous recombination in *E.coli* BJ5183. In vitro and in vivo studies indicate that the vaccine demonstrates excellent safety and immunogenicity, promising for preventing acute respiratory diseases caused by HAdV-3. Additionally, it may serve as a model for developing vaccines targeting other important serotypes of adenovirus respiratory pathogens [217]. Non-pathogenic avian adenoviruses possess the largest genome, containing an amount of non-essential DNA sequences. These have been used in studies on virus-host interactions and viral gene functions [218–221]. Pei et al. constructed the first infectious clone encompassing the entire genome of the non-pathogenic avian adenovirus 4. By targeting the deletion of ORF 16 and 17 in the pathogenic avian adenovirus 4 ON1 region and replacing them with EGFP expression cassette, a recombinant virus with reduced replication capacity was generated. The authors further investigated the differential expression of the foreign gene in this genomic region by generating EGFP cassettes in two orientations, and the results indicated that the CMV promoter in the leftward orientation might negatively affect the expression of viral genes, promoting wild-type replication levels, providing insights for future studies on viral gene functions and vector design [222].

## Challenges and future directions in viral infectious clone technology

The development of viral infectious clones based on yeast or bacterial artificial chromosome platforms has significantly advanced reverse genetics technology, providing reliable tools for vaccine development, antiviral drug screening, pathogenesis and virulence studies, as well as gene therapy and vector design. However, some challenges, such as technical limitations, ethical considerations, and regulatory hurdles, must be resolved to maximize the translational impact of this technology.

### Technical limitations

Although YAC, BAC, and YAC-BAC technologies have been applied to rescue various viruses, there are several technical challenges in the construction and application of virus infectious clones. Firstly, issues related to the fidelity of gene sequences may arise during the process of obtaining template fragments. To mitigate these issues, the optimization of PCR amplification conditions and the use of stepwise synthesis methods can reduce the error rate of PCR and improve sequence accuracy. Secondly, as the construction process occurs in host cell, mutations are often unavoidable during the assembly of full-length cDNA. Insertional and deletion mutations can be detected using restriction fragment length polymorphism analysis, but this method is only applicable to high-concentration plasmids. Point mutations must be verified through genomic sequencing. Moreover, different construction strategies, the selection of exogenous genes, and the modification site can influence the stability of the recombinant virus and lead to biological characteristics changes [223, 224]. Therefore, careful consideration of these factors and their optimization using appropriate strategies is important during the preliminary design phase [225–227]. Although YAC, BAC, and YAC-BAC technologies enable the rapid synthesis of infectious clones, genomic modification of the clone remains cumbersome even after an infectious clone is obtained. To address this question, CRISPR-Cas and other technologies can be employed to directly modify the infectious clone, enabling the rapid generation of genetically modified recombinant viruses [228, 229]. The CRISPR technology is a well-established gene editing tool [230], with the precise targeting, cleavage, and repair of specific DNA sequences, it facilitating efficient and accurate genome editing [231]. Currently, the application of CRISPR technology in DNA virus genome editing has made great progress, especially for the herpesviruses and adenoviruses [232]. However, the off-target effects [233] limited its application for RNA viruses. Nevertheless, recent studies combined CRISPR technology with de novo synthesis techniques of YAC and BAC can effectively edit RNA virus infectious clones [122, 234]^.^. This progress provides new technological approaches for RNA virus research and is expected to promote therapeutic strategies against RNA virus-related diseases.

### Future advancements

The advancement of synthetic biology has greatly propelled research in virology. High-fidelity polymerases and continuously evolving vectors have significantly reduced the limitations associated with constructing infectious clones. The outbreak of SARS-CoV-2 has further accelerated the development of emerging technologies such as Circular Polymerase Extension Reaction (CPER) and Infectious Subgenomic Amplicons (ISA). These PCR-based methods allow for the easy and rapid manipulation of viral genomes without the need for bacterial or yeast cloning [27, 28, 235]. However, challenges remain, including lower rescue efficiencies [55] additional steps of virus passage and sequence confirmation [93].

TAR technology based on yeast artificial chromosomes (YAC) and bacterial artificial chromosome (BAC) technology has been widely applied. The YAC-BAC infectious clone construction technique, which combines the advantages of YAC and BAC technologies, enables the rapid acquisition and modification of viruses in a short time frame. This approach provides a new way for the study of large and highly pathogenic viruses. It offers a robust platform for investigating viral genome functions, understanding the pathogenesis of viral infections, and developing vaccines and antiviral drugs. YAC and BAC technologies have been used in the areas of large-scale viral genome libraries and multi-virus systems [223, 236]. The capacity to accommodate large fragments of exogenous DNA facilitated the assembly of complex viral genome libraries [237], as well as the functional analysis of interactions and dynamics within multi-virus systems [238].

Currently, YAC-BAC technology has been widely applied to the rescue of herpesviruses, baculoviruses, coronaviruses, and flaviviruses. We believe that this modular assembly method can serve as a universal platform for other large dsDNA viruses and RNA viruses, with potential further applications in negative-strand RNA viruses.

### Ethical and regulatory considerations

As a classic technique of reverse genetics, infectious clones allow the de novo synthesis and assembly of viruses from their genomes, facilitating related research and applications. Advances in this technology have also raised concerns about the potential environmental release of synthetic organisms and viruses [2]. Since the first synthetic DNA genome was created, bioethics and biosafety issues have become integral to the field of synthetic genomics. In 2010, the U.S. Department of Health and Human Services (HHS) issued guidelines for commercial gene synthesis providers, which included sequence screening of orders and customer screening. Later, numerous committees, boards, and review panels have discussed various issues related to synthetic biology, providing action items and proposed regulations [239–241] Corresponding procedures have also been implemented to prevent the assembly of unapproved pathogens at the design stage [242]. Beyond regulatory measures, researchers should incorporate watermarks into synthetic genomes to prevent confusion with naturally occurring organisms and take additional measures to limit the survival capacity of synthetic organisms to laboratories and production facilities to mitigate the risk of accidental release.

## Data Availability

Not applicable.
